# Neural Compensatory Response During Complex Cognitive Function Tasks in Mild Cognitive Impairment: A Near-Infrared Spectroscopy Study

**DOI:** 10.1155/2019/7845104

**Published:** 2019-06-19

**Authors:** Jin A. Yoon, In Joo Kong, JongKwan Choi, Ji Yeong Baek, Eun Joo Kim, Yong-Il Shin, Myoug-Hwan Ko, Yong Beom Shin, Myung Jun Shin

**Affiliations:** ^1^Department of Rehabilitation Medicine, Pusan National University School of Medicine and Biomedical Research Institute, Pusan National University Hospital, Republic of Korea; ^2^OBELAB Inc., Republic of Korea; ^3^Department of Neurology, Pusan National University School of Medicine and Biomedical Research Institute, Pusan National University Hospital, Republic of Korea; ^4^Department of Rehabilitation Medicine, Pusan National University School of Medicine, Pusan National University Yangsan Hospital, Republic of Korea; ^5^Department of Physical Medicine and Rehabilitation, Research Institute of Clinical Medicine of Chonbuk National University-Biomedical Research Institute of Chonbuk National University Hospital, Republic of Korea

## Abstract

The present pilot study was aimed at conducting a comparative analysis of the level of activation in the prefrontal cortex among a normal elderly group and amnestic and nonamnestic mild cognitive impairment (MCI) groups and investigating the presence of neural compensatory mechanisms according to types of MCI and different cognitive tasks. We performed functional near-infrared spectroscopy (fNIRS) along with cognitive tasks, including two-back test, Korean color word Stroop test, and semantic verbal fluency task (SVFT), to investigate hemodynamic response and the presence of neural compensation and neuroplasticity in the prefrontal cortex of patients with amnestic and nonamnestic MCI compared with a healthy elderly group. During the two-back test, there was no significant difference in the bilateral region-of-interest (ROI) analysis in the three groups. During the Stroop test, right-sided hyperactivation compared to the left side during the task was shown in the nonamnestic MCI and normal groups with statistical significance. Mean acc*∆*HbO_2_  on the right side was highest in the nonamnestic MCI group (0.30 *μM)* followed by the normal group (0.07 *μM*) and the amnestic MCI group (-0.10 *μM*). Otherwise, intergroup ROI analysis of acc*∆*HbO_2_ in these activated right sides showed no significant difference. During the VFT test, there was no significant difference in the bilateral region-of-interest analysis in the three groups. The highest mean acc*∆*HbO_2_ was shown in the normal group (0.79 *μM*) followed by the nonamnestic MCI group (0.52 *μM*) and the amnestic MCI group (0.21 *μM)*. Otherwise, there was no significant difference between groups. The hemodynamic response during fNIRS showed different findings according to MCI types and cognitive tasks. Among the three tasks, the Stroop test showed results that were suggestive of neural compensatory mechanisms in the prefrontal cortex in nonamnestic MCI.

## 1. Introduction

Functional brain activity during a cognitive task is one of the most important biomarkers for the early diagnosis of Alzheimer's disease (AD) and mild cognitive impairment (MCI). Several studies have examined the hemodynamic response on functional near-infrared spectroscopy (fNIRS) when conducting various neuropsychological batteries [1–3]. In particular, when conducting such assessments for diagnosing AD, longitudinal episodic memory and semantic verbal fluency tests (VFTs) are considered appropriate tools for evaluating progressive cognitive deterioration; however, the results of previous studies have shown no meaningful decrease in the hemodynamic response of the prefrontal cortex or lateralization of patients with MCI, apart from the results for AD [4–6]. There have been attempts to confirm the hemodynamic response of the prefrontal cortex through working-memory loading by conducting verbal *n*-back tests [4, 5], VFTs, and Stroop tests as executive function tests [6]. However, no previous study has compared the results of neuropsychological tests for which the hemodynamic response, as indicated by fNIRS signals, is more sensitive, according to the types of MCI; further, various tests have not been conducted on the same patient. We expect that functional brain activity during various cognitive tasks would increase the diagnostic sensitivity for MCI. In a recent study by Yap et al. [7], the activation of the bilateral prefrontal cortex during a VFT was observed to be greater in the MCI group than in the normal group; this finding was explained by the recruitment of additional circuitry through neuroplasticity and a neural compensation mechanism. Therefore, by confirming the pattern of hemodynamic response during different tasks according to the types of MCI, it may be possible to suggest a useful biomarker for the early diagnosis of MCI through fNIRS and for monitoring disease progression. Currently, diagnosis of MCI and AD relies on patients' clinical evaluations [8–10]. In addition, establishment of the relationship of metabolic activity with structural changes and amyloid plaque load by conducting functional magnetic resonance imaging (fMRI) [7] and positron emission tomography (PET) [11] would be helpful to reduce the variability of diagnostic outcome and to distinguish the MCI group from the AD group. Nonetheless, the cost of these techniques makes them difficult to be utilized as routine clinical tools. Further fNIRS studies are needed to obtain additional results following the accurate selection of patient groups and the subdivision of the MCI group. Therefore, the present study was aimed at conducting a comparative analysis of the level of activation in the prefrontal cortex among a normal elderly group and amnestic and nonamnestic MCI groups and investigating the presence of neural compensatory mechanisms according to types of MCI and different cognitive tasks.

## 2. Materials and Methods

### 2.1. Study Participants

Twelve normal elderly people and 15 patients with MCI aged more than 65 years were recruited based on the criteria proposed by Petersen et al. [12]. Final diagnosis was confirmed by a neurologist using a multidisciplinary approach, including medical examination, neuropsychological and neuroimaging assessments, and neurocognitive tests, such as the Seoul Neuropsychological Screening Battery 2nd edition, brain MRI, and F-18 (flutemetamol) amyloid PET/computed tomography. The measurement tools used to allocate the participants to the normal and amnestic and nonamnestic MCI groups are shown in Table 1; this was followed by application of the participant selection criteria. This study was approved by the Institutional Review Board of our hospital (approval number: 1809-019-071). Patients were enrolled in the study after they had provided written informed consent.

### 2.2. Experimental Design

**Figure 1:**
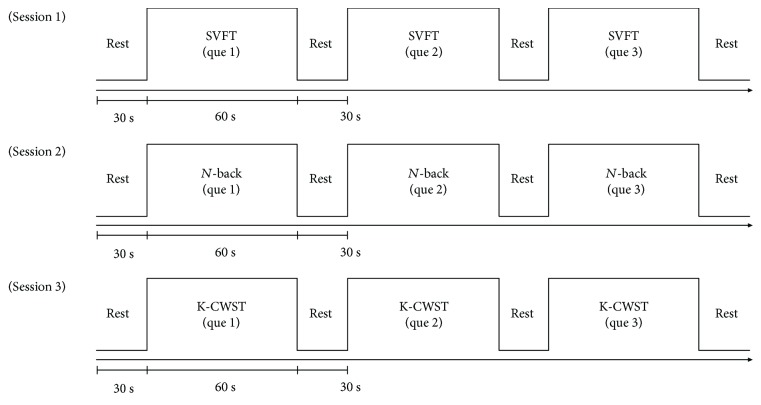
Cognitive task protocol used for the NIRSIT system. K-CWST: Korean color word Stroop test; SVFT: semantic verbal fluency task.

According to the result of the neuropsychological tests, we divided the patients into three groups: normal (*n* = 12), amnestic MCI (*n* = 9), and nonamnestic MCI (*n* = 6). Each group performed the *n*-back and Stroop tests and VFT. Each task was performed thrice, and a 30 s break was provided between tasks. One type of task was conducted per session, and a 5 min break was provided between each session. The protocol is shown in Figure 1.

We recorded the hemodynamic response during the performance of the protocol using a commercial wireless continuous-wave near-infrared spectroscopy system (NIRSIT; OBELAB Inc., Seoul, Republic of Korea) [13, 14]. The cognitive tasks performed during this study were as follows:

*(1) *n*-Back Task (Two-Back Task) [15]*. The *n*-back task was performed for working memory assessment. The examiner presents the words while the participant performs the working memory, and the number of memorized words is measured afterward. In this task, participants decide whether a stimulus presented as part of a sequence matches the one that was presented *n* items ago. The number of correctly memorized/identified stimuli is measured.

*(2) Korean Color Word Stroop Test [16]*. The Stroop test was performed to measure mental control and response flexibility. This task measures new responses elicited while suppressing the dominant response. The tasks often involve letter reading, color reading, and so on. In this study, the task involved reading the color of a letter written in red, blue, yellow, or black within a limited time frame.

*(3) Semantic Verbal Fluency Task (SVFT) [17]*. An SVFT involves generating as many words as possible within a certain time frame and hints at the semantic category of the words generated. The task measures the amount of information regarding categorization and the number of words that can be retrieved from memory in 1 min. In this study, the task involved generating as many words as possible in relation to the keywords.

### 2.3. Data Processing

**Figure 2:**
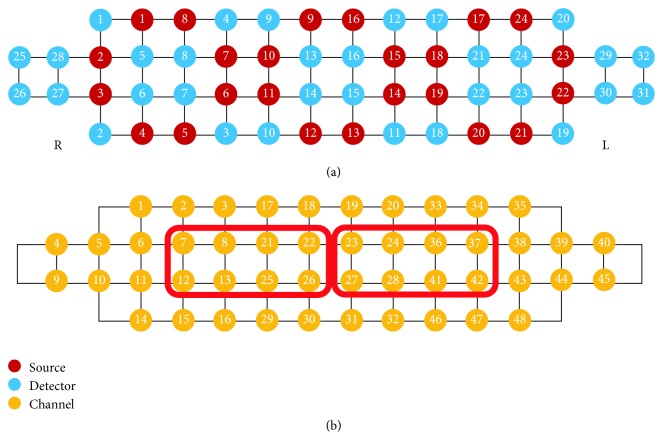
Arrangement of sources and detectors and location of region-of-interest channels.

Hemodynamic response of the prefrontal cortex was recorded using a high-density NIRS device (NIRSIT; OBELAB Inc.), which was composed of 24 sources (laser diodes) emitting two wavelengths (780/850 nm) and 32 photodetectors, at a sampling rate of 8.138 Hz [18]. The unit distance between the source and the detector was 1.5 cm; the source-detector array is shown in Figure 2(a). In this study, only 3 cm channels were analyzed and the channel configuration is shown in Figure 2(b).

The total number of channels was 48, and the detected light signals in each wavelength were filtered by a band-pass filter (0.005–0.1 Hz) to minimize environmental noise-related light and physiological noise due to body movement. The poor-quality channels (signal − to − noise ratio < 30 dB) were rejected before extraction of hemodynamics data to prevent misinterpretation. Relative hemodynamic changes of each channel during each trial of the tasks were calculated separately using the modified Beer-Lambert law [19]. The multiple trial results were block-averaged individually before grand-averaging for each group. The accumulated oxygenated hemoglobin (oxy-Hb/HbO_2_) values (acc*∆*HbO_2_) during the task period represented the activation of the prefrontal cortex.

### 2.4. Statistical Analysis

The representative means and standard deviations of acc*∆*HbO_2_ were calculated from the region of interest (ROI) of the right and the left prefrontal cortex. The right and left ROI are composed of channels 7, 8, 12, 13, 21, 22, 25, and 26 and channels 23, 24, 27, 28, 36, 37, 41, and 42, respectively. Within-group difference between acc*∆*HbO_2_ of the right and left ROI was evaluated using Wilcoxon signed-rank tests since the number of aMCI and MCI groups was not enough, and the normal group was not normally distributed after evaluating the normality of the data by Shapiro-Wilk test. Between-group differences of acc*∆*HbO_2_ were evaluated by Kruskal-Wallis tests. All statistical analyses were performed using IBM SPSS Statistics 21 (SPSS Inc., Chicago, IL, USA). The criterion for statistical significance was set at *p* < 0.05.

## 3. Result

The average age of the normal group was 67.75 ± 5.65 years old; the amnestic MCI group, 66.88 ± 6.95 years old; and the nonamnestic group, 68.37 ± 6.54 years old, with no significant differences (*p* > 0.05) between groups on age, sex ratio, or education level.

**Figure 3:**
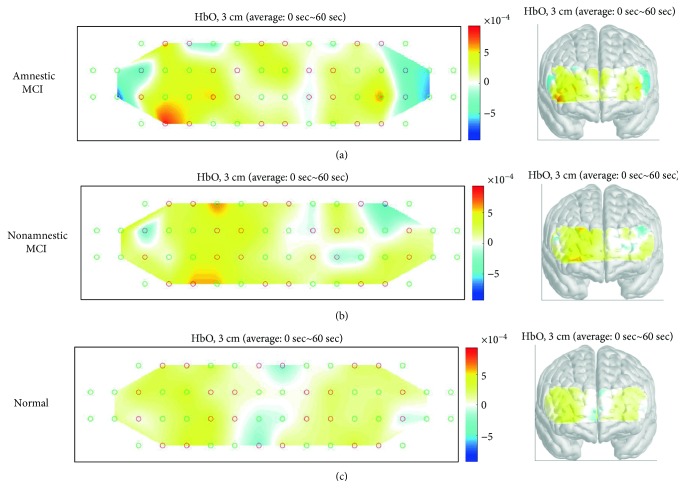
Activation map during the two-back test in three groups showing no significant difference between groups. MCI: mild cognitive impairment.

**Figure 4:**
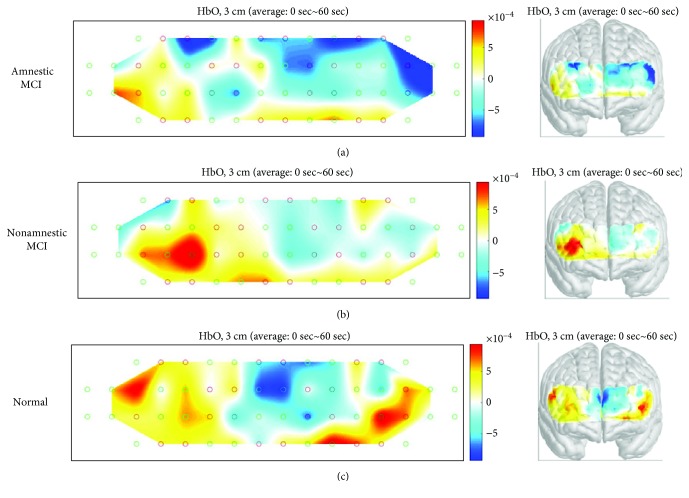
Activation map during the Stroop test in three groups showing hyperactivation of the right prefrontal cortex in the nonamnestic MCI group compared to the normal group. MCI: mild cognitive impairment.

**Figure 5:**
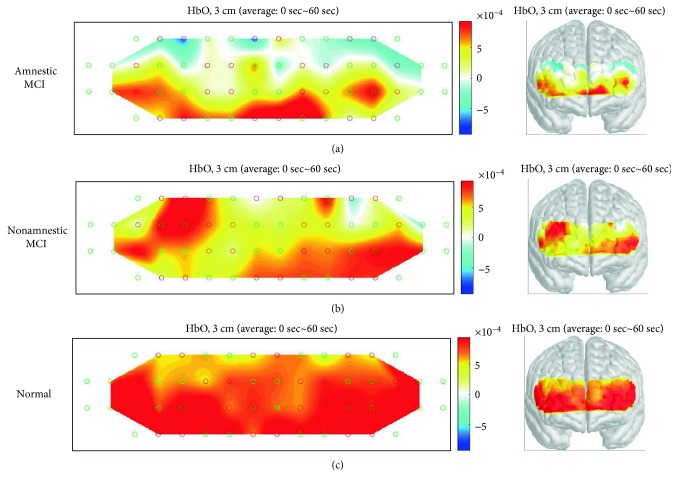
Activation map during the verbal fluency test in the three groups showing different hemodynamic responses by groups. MCI: mild cognitive impairment.

**Figure 6:**
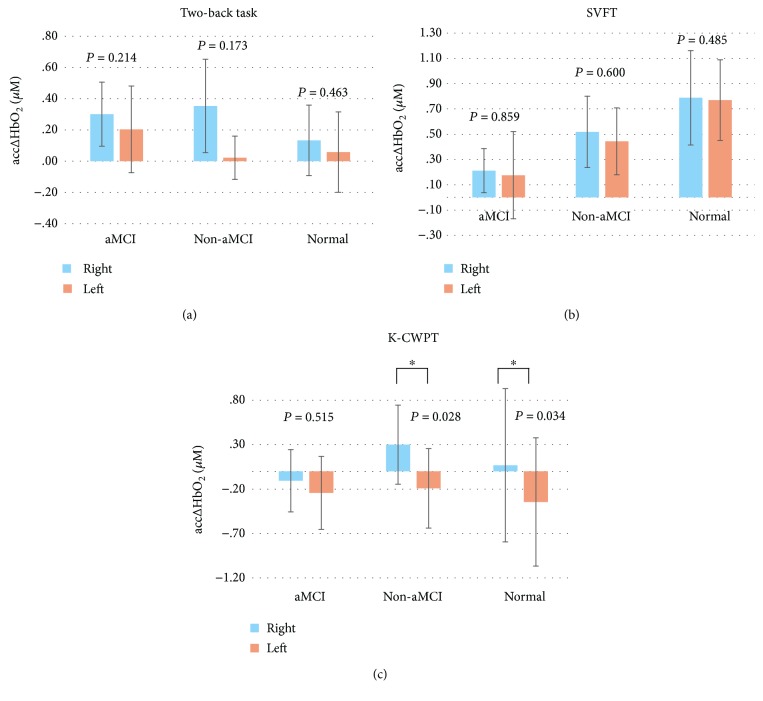
Mean acc*∆*HbO_2_ of bilateral prefrontal regions during the tasks in the three groups.

The hemodynamic response during different cognitive tasks was shown as illustrated in Figures 3–5. During the two-back test, there was no significant difference in bilateral region-of-interest (ROI) analysis (channels 7, 8, 12, 13, 21, 22 ,25, and 26 present the right prefrontal region; channels 23, 24, 27, 28, 36, 37, 41, and 42 present the left prefrontal region) (Figure 2) in three groups. During the Stroop test, the change in oxy-Hb concentration during the activation period by the group was shown as illustrated in Figure 4. Right-sided hyperactivation compared to the left side during the task was shown in the nonamnestic MCI and normal groups with statistical significance. Mean acc*∆*HbO_2_ on the right side was highest in the nonamnestic MCI group (0.30 *μM)* followed by the normal group (0.07 *μM*) and the amnestic MCI group (-0.10 *μM*). Otherwise, intergroup ROI analysis of acc*∆*HbO_2_ in these activated right sides showed no significant difference (*p* = 0.534). During the VFT test, there was no significant difference in the bilateral region-of-interest analysis in three groups. The highest mean acc*∆*HbO_2_ was showed in the normal group (0.79 *μM*) followed by the nonamnestic MCI group (0.52 *μM*) and the amnestic MCI group (0.21 *μM)*. Otherwise, there was no ignificant difference between groups (Figure 6).

## 4. Discussion

The two key findings of our study is the dominant right prefrontal cortex lateralization during the Stroop test in the nonamnestic MCI and normal groups and higher activation of right PFC in the nonamnestic MCI group compared to the normal group during the Stroop test. Although the latter one showed no statistical significance due to limited subjects, higher mean acc*∆*HbO_2_ in the nonamnestic MCI group during the Stroop test task is obvious as illustrated in the activation map. A previous study, which explained the concept of neuroplasticity, has reported that the MCI subjects show hyperactivation in the right prefrontal cortex to maintain cognitive function [10]. In our study, higher hemodynamic response in the right prefrontal cortex compared to the left side was found in the nonamnestic MCI and the normal group which can also be explained as compensatory effect to supplant left prefrontal function. Amnestic MCI, with episodic memory impairment, has been identified as a possible precursor of AD [20]. The prefrontal cortex, especially that on the right side, is involved in the formation of new episodic memories. In the amnestic MCI group, findings in our study with no significant lateralization in the right prefrontal cortex during the Stroop test could be explained as compromised compensatory mechanism compared to the other groups.

Although the neuroplasticity of the right prefrontal cortex in MCI has been described earlier, this is the first study to compare its effect between amnestic and nonamnestic MCI groups preforming various cognitive tasks.

Inconsistent results have been obtained for brain activation, as detected on fMRI, in amnestic MCI during various executive tasks. In nonamnestic MCI, deficits other than that in the memory domain are dominant, and cognitive impairments, including memory deficits, are described as amnestic MCI. An fMRI study showed that there are no areas in which the amnestic MCI group showed more activation than the nonamnestic MCI group during encoding and recognition [21]. To date, there are no fNIRS studies to compare the findings between amnestic and nonamnestic MCI. According to the results of our study, the neuroplasticity of the right prefrontal cortex during the Stroop test was preserved only in the nonamnestic MCI group. According to the result of our pilot study, the Stroop test might be the most sensitive task to discriminate between these two groups.

Based on the result of previous studies [22, 23], our finding can be explained as compensatory activation in the right prefrontal cortex, with higher activation in the nonamnestic MCI group than in the normal group. As this is the first study to compare sensitivity of detecting neural compensation during various cognitive tasks, the results of our test can be explained in several ways. First, the Stroop test might be a sensitive tool to evaluate cognition flexibility impairment, which has shown strong relationship with episodic memory in a previous study [24]. Our finding of hypoactivation in the amnestic MCI group and compensatory hyperactivation in the nonamnestic MCI group on the right prefrontal cortex can be explained with this approach. The cognitive flexibility impairment could sufficiently be compensated for in the nonamnestic MCI group but not in the amnestic MCI group. The Stroop test could be used for evaluating cognitive control and preservation of neural compensatory mechanisms in MCI, and therefore, further studies with large sample sizes should be performed. Further, the two-back test showed no statistically significant or visible difference in the activation map among the three groups. To extend the results of a previous study which showed no difference in performance during the two-back task between healthy elderly individuals [6], this task might be insufficient to distinguish differences in prefrontal cortical activation, including neural compensation during working memory processing in patients with MCI. Although there was no difference in performance during two-back and three-back tasks in a previous study [6], additional trials divided by types of MCI like our study should be performed laterally to define this task as insufficient working load.

The limitation of our study is the small sample size. However, the finding of our pilot study suggests the possibility of meaningful fNIRS findings in patients with MCI. Further studies with larger sample sizes should be conducted including patients with MCI and AD.

## 5. Conclusions

The hemodynamic response, as detected on fNIRS, differs according to MCI types and cognitive tasks. Among the three tasks used in this study, the Stroop test was the suitable tool for detecting neural compensatory mechanisms in the prefrontal cortex. Task-oriented neural compensation was not observed in the amnestic MCI group.

## Figures and Tables

**Table 1:** Tests performed for application of the participant inclusion criteria.

Neuropsychologic assessment	Clinical dementia scale
	Dementia activity of daily living
	Mini mental state examination
	Dementia neuropsychiatric inventory
	SNSB II

Neuroimaging assessment	Brain MRI
	F-18 (flutemetamol) amyloid PET/CT

SNSB II: Seoul Neuropsychological Screening Battery 2nd edition; MRI: magnetic resonance imaging; PET: positron emission imaging; CT: computed tomography.

## Data Availability

The data used to support the findings of this study are restricted by the Institutional Review Board of Pusan National University Hospital in order to protect patient privacy. Data are available from Jin A. Yoon, yjk5289@naver.com, for researchers who meet the criteria for access to confidential data.
